# SENS vs. the hallmarks of aging: competing visions, shared challenges

**DOI:** 10.1007/s10522-025-10248-5

**Published:** 2025-05-05

**Authors:** Pablo García-Barranquero, Saúl Pérez-González

**Affiliations:** 1 University of Malaga, Málaga, Spain; 2 University of Valencia, Valencia, Spain

**Keywords:** Aging, Hallmarks of aging, Geroscience, Mechanism, SENS

## Abstract

Aging research is often framed within pluralistic frameworks that emphasize cellular and molecular damage processes. Among the most influential are Strategies for Engineered Negligible Senescence (SENS), which aims to counteract biological decline through targeted damage repair, and the Hallmarks of Aging (HoA), which seeks to identify fundamental mechanisms underlying this process. Both proposals, although diverse, significantly influence contemporary approaches to the challenges posed by aging. However, despite extensive discussion, we contend that key conceptual and methodological aspects remain insufficiently explored. This paper seeks to advance the debate by critically analyzing and comparing their foundational goals, theoretical premises, and research frameworks. Specifically, we examine their definitions of aging, perspectives on health and disease, approaches to scientific evidence and causal interventions, and communications strategies. In doing so, we aim to contribute to a deeper understanding and more nuanced assessment of both SENS and the HoA.

## Introduction

For a long time, aging was explained through philosophical, religious, and mystical perspectives (Gruman 1956). In the twentieth century, however, evolutionary theories such as mutation accumulation (Medawar 1958), antagonistic pleiotropy (Williams 1957), and the disposable soma theory (Kirkwood and Holliday 1979) became dominant. Although these theories explained why aging occurs, they could not account for how it works. This limitation led to a shift toward mechanistic explanations of aging that, with advances in molecular biology and genetics, gave rise to monocausal theories like oxidative stress (Harman 1956), telomere shortening (Blasco 2005), and mitochondrial DNA mutations (Wallace 1992). Nevertheless, recent research has shown that aging results from multiple interconnected processes (de Grey et al. 2002; López-Otín et al. 2013; Kennedy et al. 2014; Schmauck-Medina 2022).

In response to the complexity of aging, a range of pluralistic frameworks have emerged to address its challenges. Two particularly relevant proposals in this regard are Strategies for Engineered Negligible Senescence (SENS) (de Grey et al. 2002; de Grey 2023), which advocates repairing accumulated damage in the body to reverse its effects, and the Hallmarks of Aging (HoA) (López-Otín et al. 2013; 2023), which identifies and classifies the key biological processes driving age-related decline.^1^ Given their prominent roles, both SENS^2^ and the HoA are the subject of ongoing debate and scrutiny. SENS has been criticized regarding the burden of proof, an epistemological principle stipulating that those who propose a novel hypothesis must provide robust evidence to substantiate it. Le Bourg (2013) argues that, while SENS offers theoretically compelling proposals, it lacks empirical validation demonstrating its feasibility in complex organisms. Furthermore, Rattan (2020) notes that, although aging is characterized by the stochastic accumulation of molecular damage, the intrinsic capacity of organisms to sustain homeostasis through repair and maintenance mechanisms is inherently constrained. The HoA has also faced considerable criticism, particularly for lacking a clear framework to prioritize therapeutic targets. Gems and de Magalhães (2021) argue that the absence of a defined hierarchy makes it difficult to identify which processes should be targeted first in clinical interventions. They challenge the assumption that cellular damage is the primary driver of aging, suggesting its role may not be as critical as commonly believed. Additionally, Keshavarz et al. (2023) emphasize that therapies need to integrate the complex interactions between aging regulators to effectively slow the aging process and improve clinical outcomes.

We contend that, despite their divergences, a comparative analysis of the conceptual and methodological foundations of SENS and the HoA is both feasible and valuable. There are several considerations that support the relevance of such analysis.^3^ First, SENS and the HoA rest on a remarkably similar theoretical foundation: both conceptualize aging as a multifactorial phenomenon driven by interconnected biological mechanisms, primarily at the cellular and molecular levels. In this regard, de Grey (2023) has compared the two approaches, arguing that their respective categories exhibit strong conceptual alignment. Second, both proposals have played a pivotal role in shaping the idea that aging is a plastic process—one that, in principle, can be modulated through biotechnological interventions—insofar as it is conceived as amenable to targeted manipulation (see also, Campisi et al. 2019; Kennedy et al. 2014). Third, while sharing the overarching goal of addressing aging, SENS and the HoA have engaged in a form of intellectual and material competition, seeking to influence the conceptual direction and the allocation of recourses in aging research (Duque 2023). Finally, both proposals have achieved sustained prominence. Although recognized in different contexts, each has exerted considerable influence: SENS has garnered widespread public and media attention through its provocative vision of the imminent defeat of aging (García-Barranquero and Bertolaso, 2022), while the HoA has become a widely cited and authoritative reference within the geroscience community, increasingly regarded as a foundational paradigm in the field (Gems and de Magalhães 2021).

By means of a comparative analysis, this article aims to deepen the discussion on SENS and the HoA and uncover relevant similarities and differences that extend beyond those commonly addressed in the literature. While acknowledging existing debates, we consider that important conceptual and methodological aspects of these frameworks remain underexplored. Our work seeks to address this gap and, in doing so, to encourage a more comprehensive discussion of research frameworks in the study of aging. To structure this inquiry, we will begin by clearly outlining the scope and objectives of each framework, emphasizing the foundational aims and the issues they seek to address ("Scope and aim" section). We will then critically examine their conceptualization of aging ("Conceptualization of aging" section). Following this, we will explore the relationship between aging, health, and disease, engaging with the ongoing debate about whether aging should be regarded as a pathological condition ("Health and disease" section). Next, we will assess how each framework approaches causal interventions and scientific evidence ("Interventions and evidence" section). We will then analyze how these ideas and proposals are communicated within scientific and public discourse ("Communication" section). Finally, we will offer some general conclusions ("Conclusions" section).

## Scope and aim

Aging has long been recognized as a process that not only limits lifespan but also progressively undermines health and functionality. While once viewed as an inevitable biological fate, contemporary research increasingly frames it as a challenge that can—and should—be addressed through targeted interventions.^4^ The detrimental effects of aging, ranging from chronic diseases to reduced quality of life, have profound social and economic consequences, reinforcing the urgency of developing strategies to mitigate its impact. In this context, both SENS and the HoA approach aging from an applied standpoint, aiming to devise interventions that improve health outcomes. However, despite their shared ultimate aim, their methodologies and objectives differ significantly, with SENS advocating for the reversal of aging and the HoA focusing on optimizing biological function and extending healthspan.

SENS adopts an explicitly interventionist stance, rooted in technological solutionism—an engineering-driven perspective that treats aging as a technical problem requiring practical solutions rather than deeper theoretical understanding. In this view, if a solution does not yet exist, it is only because science is not yet advanced enough to develop it, but progress will inevitably provide the necessary tools.^5^ Rather than aiming to slow aging, SENS seeks to reverse its effects, operating on the premise that repairing damage is more straightforward than merely delaying its accumulation. SENS initially considered nine categories of damage (e.g., cell loss, cell senescence) (de Grey et al. 2002), although they were subsequently reduced to seven upon integrating endocrine aging and immune aging into the other categories (de Grey 2023).^6^ Crucially, its focus is not on understanding how aging occurs but on how to fix it—identifying key forms of structural and functional deterioration and counteracting them through biomedical interventions, including gene therapies, stem cell treatments, and regenerative medicine strategies. Aging, in this framework, is not an inevitable decline but a maintenance issue: with periodic repairs, its effects can be minimized or undone (de Grey 2008). Inspired by engineering and preventive maintenance models, SENS aspires to create iterative treatments that provide temporary life extension—such as therapies adding a few extra years—while continuously refining and enhancing interventions to push longevity further with each successive breakthrough; this concept is often referred to as the *longevity escape velocity* (de Grey 2004). SENS understands rejuvenation as the restoration of the body to a state of peak biological function, free from the accumulated damage of aging, enabling individuals to function at their highest potential.

The HoA, by contrast, adopts a more descriptive and explanatory approach, aiming first to understand the biological foundations of aging before attempting to modify them. Rather than framing aging as a repairable dysfunction, the HoA conceptualizes it as a complex, emergent phenomenon arising from multiple interconnected processes. This approach, grounded in an extensive review of prior studies, offers a cohesive theoretical framework that advances our understanding of aging. By outlining key hallmarks—such as genomic instability, mitochondrial dysfunction, and cellular senescence—the HoA provides a structured framework for studying its underlying mechanisms (see "Introduction" section). In the revised version of the HoA (López-Otín et al. 2023), alongside the addition of three new hallmarks (autophagy dysfunction, chronic inflammation, and microbiota alterations), there has been a concerted effort to update and expand the understanding of the molecular and cellular mechanisms involved in aging. Other modifications include a stronger emphasis on the interaction between the hallmarks, an updated focus on cellular senescence, a deeper exploration of DNA repair and telomeres, and an increased attention to cellular regeneration and tissue repair. These updates provide a more integrated and dynamic perspective on aging, recognizing the interdependence of biological mechanisms driving the process. Although it does not propose a specific interventionist roadmap, the HoA aims to provide a foundation for the development and evaluation of therapeutic strategies. Indeed, in assessing potential hallmarks, the HoA considers available interventions targeting age-related damage. It should be noted, however, that the HoA primarily focuses on modulating biological processes to optimize function, ultimately aiming to extend healthspan rather than radically prolong lifespan (Tartiere et al. 2024).

Despite their differences, SENS and the HoA contribute to the broader discourse on aging interventions. While SENS prioritizes repair and rejuvenation, the HoA aims to refine our understanding of aging mechanisms to guide more effective therapies. These approaches—one focused on direct action, the other on fundamental knowledge—highlight the ongoing debate in geroscience over whether aging should be treated as a solvable engineering problem or a complex biological phenomenon.

## Conceptualization of aging

In recent years, the issue of defining aging and reaching a consensus within the geroscientific community has gained significant prominence (see Golubev 2021; Guillermain 2022; Lemoine 2020). Traditionally, aging has been understood as a natural, normal, and inevitable process. This traditional view framed aging as an intrinsic characteristic of all living organisms, a phenomenon that occurs without external intervention and inevitably leads to physical and functional decline over time (cf. Hayflick 2007). Aging was thought to be an irreversible process, with no possibility of stopping or reversing it. However, advances in science and technology have increasingly challenged this view, suggesting that aging is not a fixed phenomenon, but one that can be influenced, modified, and potentially reversed through biological and technological means (Blasimme 2021; Saborido and García-Barranquero 2022). SENS and the HoA both embrace this view and aim to actively address aging, rather than merely accepting it. However, both proposals differ on how much aging can be modified and whether it is reversible. These differences shape their conceptualization of aging; determining whether it should be approached as a process to be managed, repaired, or fundamentally altered.

As previously discussed (see "Scope and aim" section), SENS adopts an explicitly interventionist stance rooted in a technological solutionist perspective, framing aging as a technical problem that necessitates practical solutions rather than a deeper theoretical understanding.^7^ It does not provide a strict definition of aging, as its main focus is on the effectiveness of treatments rather than conceptual debates. De Grey (2013) argues that a precise definition of aging is unnecessary from a biomedical perspective, emphasizing the importance of focusing on the repair and prevention of accumulated damage. From this standpoint, semantic debates are not only unproductive but also delay the development of effective interventions (de Grey et al. 2002).^8^ Rather than seeking to explain why aging occurs, SENS is fundamentally concerned with mitigating its effects through targeted biomedical interventions.

The HoA explicitly defines aging, as its approach necessitates a clear conceptualization to integrate and systematize the existing body of biological knowledge. Aging is described as a “progressive deterioration of physiological integrity, leading to impaired function and increased vulnerability to death” (López-Otín et al. 2013). Unlike SENS, which emphasizes intervention over definition, the HoA regards a precise understanding of aging as essential for identifying the key biological processes that underlie its progression. In defining aging, the HoA establishes a cohesive framework that guides future research and interventions, countering the view that aging is too complex or ill-defined to be studied systematically. This definition thus provides a structured framework for understanding aging as a measurable, progressive biological phenomenon.

## Health and disease

As discussed above, SENS and the HoA both acknowledge that aging undermines health and functionality (see "Scope and aim" section). They link aging to various physical and mental challenges, and recognize its impact on capabilities and quality of life. However, SENS and the HoA diverge in their conceptualization of the relationship between health and disease, and their views on the pathologization of aging.

SENS aligns with the ideal model of health and disease, which has traditionally been prevalent among both lay audiences and healthcare professionals. This model defines health strictly as the absence of disease, establishing a binary framework in which health and disease are mutually exclusive and collectively exhaustive—everyone is either healthy or diseased (Hofmann 2005). Following this logic, SENS conceptualizes aging as a disease because it progressively undermines health by impairing normal biological functions. It views aging as an inherently negative, pathological process, responsible for the gradual deterioration of the organism at the cellular and molecular levels. In fact, aging is not only seen as a condition in itself, but also as the primary driver of many chronic and degenerative diseases, such as cancer, cardiovascular diseases, and neurodegenerative disorders. This challenges the conventional distinction between aging and disease, framing the former as an overarching pathological process rather than a mere background for age-related conditions (de Grey 2004).

On the other hand, the HoA aligns with the medical model of the relationship between health and disease. The medical model, which is close to medical practice, holds that disease goes beyond the absence of health (Hofmann 2005). Although necessary, the absence of health alone is insufficient for disease; it must meet additional criteria, often defined by standardized methods and symptom descriptions. In this proposal, health and disease are considered mutually exclusive but not collectively exhaustive. While nobody can be healthy and diseased at the same time, someone can be neither healthy nor diseased. According to the medical model, and despite its detrimental effects, the HoA does not conceptualize aging as a disease. Rather, it describes aging as a series of biological changes affecting the body over time—a natural, universal process inherent to all organisms, rather than a pathological deviation affecting some but not others. This is broadly consistent with a Boorsean conception of disease as a deviation from species-typical functioning (Schramme 2013).

Moreover, in contrast to the ideal model, López-Otín and Kroemer (2021) define health not just as the absence of disease, but as a dynamic state of physiological balance reliant on the integrity of several biological systems, such as homeostasis, stress response, and adaptive capacity. Aging, from this perspective, is seen as the gradual loss of these health markers, which increases susceptibility to disease without categorizing aging itself as pathological.

The model of the relationship between health and disease, and the resulting conceptualization of aging, further illuminates some abovementioned divergences between SENS and the HoA. Given its disease nature, SENS considers aging as a pathology that can and should be corrected (or even reversed) through advanced medical interventions. There is a moral obligation to combat aging (de Grey 2005a; see also Farrelly 2022). The HoA, however, holds that strategies can be implemented to manage aging and maintain quality of life, without eliminating the aging process. The absence of aging is not required for absence of disease. From this perspective, there is no moral obligation to eliminate aging, but rather a commitment to improving health and well-being as we age, aiming to live as satisfactorily as possible (Glannon 2008; Hayflick 2007).

## Interventions and evidence

As previously mentioned, SENS adopts an engineering approach to aging, concentrating on available or foreseeable interventions aimed at repairing cellular and molecular damage. In contrast, the HoA takes a more descriptive and explanatory stance, focusing on identifying and evaluating potential hallmarks, with particular attention to the causal mechanisms linking these markers to the biological changes over time. Nonetheless, in the HoA, interventions targeting age-related damage are also considered and play a crucial role.

In the HoA, interventions provide the basis for assessing potential hallmarks of aging. The assessment of hallmarks is based on three criteria: “(1) it should manifest during normal aging; (2) its experimental aggravation should accelerate aging; and (3) its experimental amelioration should retard the normal aging process and hence increase healthy lifespan” (López-Otín et al 2013, pp. 1194–1195). While the first criterion considers the causal processes involved in normal aging, the second and third focus on interventions targeting age-related damage. For each accepted hallmark, there should be interventions capable of accelerating and retarding aging. Consequently, like SENS, the HoA framework requires the systematic review and evaluation of available interventions.^9^

There are, nevertheless, notable divergences in how SENS and the HoA assess interventions targeting age-related damage. Firstly, they differ in the severity and stringency of their evaluations. The evidence required to establish a causal claim, as well as the way the available evidence is assessed, diverge. SENS applies a less severe approach and tends to be more optimistic about the potential of interventions. For example, in line with technological solutionism, limited evidence supporting an intervention is often attributed to a lack of funding rather than to theoretical or technological problems. In contrast, the HoA adopts a more severe approach and is generally more conservative in its assessment of potential interventions. In this regard, it explicitly acknowledges concerns regarding the limited evidence supporting the efficacy of certain interventions in ameliorating aging.

Second, SENS and the HoA diverge in the types of evidence they consider when evaluating interventions. In the methodological and philosophical literature, a common distinction is drawn between evidence of correlations (or statistical evidence) and evidence of mechanisms. Evidence of correlations provides information about whether a putative cause and the effect of interest are appropriately correlated (Williamson 2019). In other words, it assesses whether differences in the putative cause are associated with differences in the effect (Illari 2011). Such evidence is typically derived from observational or experimental clinical studies, such as cohort studies, case control studies, and randomised control trials (Parkkinen et al. 2018). Evidence of mechanisms, on the other hand, provides information about the mechanisms between the putative cause and the effect of interest (Williamson 2019). It may refer to the existence of a mechanism, a property of a mechanism, a component of a mechanism, or an interaction between mechanisms (Iranzo and Pérez-González 2024). Typical sources of evidence of mechanisms include, among others, autopsies, in vitro experiments, and biomedical imaging (Parkkinen et al. 2018).

When assessing interventions, SENS primarily relies on evidence of correlations. Specifically, it reviews evidence on correlations between interventions and reductions in aging-related damage. For instance, in the case of hormone supplementation, SENS focuses on experimental studies in animal models that investigate the correlation between hormone supplementation and the mitigation of hormone secretion decline (de Grey et al. 2002). Although evidence of mechanisms is occasionally referenced, it is not regarded as essential for establishing the efficacy of an intervention. In contrast, to assess interventions, the HoA considers both evidence of correlations and evidence of mechanisms. It examines evidence of correlations between interventions targeting potential hallmarks and changes (either acceleration or retardation) in aging-related damage, alongside evidence of the mechanisms through which these interventions produce such effects. For example, in the case of target of rapamycin (TOR) inhibition, complementary evidence of the involved mechanisms is considered crucial to establish its effects (López-Otín et al. 2013). For the HoA, this kind of evidence not only supports the efficacy of an intervention but also plays a key role in identifying and evaluating potential adverse side effects.

In this respect, the HoA appears to align with pluralist approaches to evidence. Recently, medical scientists and philosophers of science have advocated for a more pluralist approach to evidence in the biomedical field (Anjum et al. 2020; Parkkinen et al. 2018; Pérez-González and Rocca 2022). They argue that evidence of mechanisms should play a more prominent role in the evaluation of causal claims. Evidence of mechanisms is considered valuable for various tasks, including clinical practice, risk assessment, and the evaluation and approval of new drugs.

## Communication

Science communication is not confined to the work of professional science communicators; rather, it also encompasses interactions between scientists and the general public, policy-makers, and even other scientists. In this broad sense, advocates of SENS and the HoA engage in a wide range of communication activities, including academic publication and public outreach. However, SENS and the HoA differ significantly in the ways they communicate their proposals.

Science communication, like science itself, is rarely value-free (Elliot 2017; Desmond 2024). There are no neutral or value-free ways of providing information; value-laden choices typically shape various aspects of science communication. Those aspects include at least frames, terms, metaphors, and categories (Elliot 2017). Consider, for instance, frames. In science communication, information is usually framed in a specific way, e.g., highlighting gains vs highlighting losses. A complete absence of framing is seldom possible or desirable (e.g., exhaustive presentation of raw data). Moreover, there is ample empirical evidence that framing can significantly influence the reception of the message and the actions it leads to. For example, gain-framed health messages are more effective than loss-framed messages (Gallagher and Updegraff 2012). Messages that highlight the benefits of engaging in a particular health behavior (e.g., smoking cessation) tend to be more effective in promoting behavior change than those that highlight the negative consequences of failing to engage. Nonetheless, framing is not value-neutral: frames embrace and support specific values and worldviews. In public health communication, for instance, a particular frame might embrace liberal values, while others may embody a communitarian approach.

In the context of science communication, both SENS and the HoA involve value-laden choices that reflect underlying aims and assumptions. Regarding framing, SENS frames aging as an urgent and solvable challenge, emphasizing the feasibility of radical life extension. It accentuates the potential benefits of interventions, such as reversing aging through mitochondrial repair or telomerase activation, while frequently downplaying or overlooking possible adverse effects. Advocates of SENS argue that negative effects are either minimal, manageable, or outweighed by the benefits, reinforcing an optimistic perspective on biomedical advancements (Zealley and de Grey 2013). The HoA, by contrast, adopts a more measured stance, balancing the promise of intervention with the recognition of biological complexity. Proponents of the HoA emphasize that targeted interventions may produce unintended consequences, necessitating careful evaluation prior to large-scale application. For instance, while telomerase activation could extend lifespan, it could also increase the risk of cancer due to uncontrolled cell proliferation (Blasco 2005).

While both SENS and the HoA consider the practical consequences of interventions, they differ in how they approach and present them. This divergence in framing is in line with the abovementioned divergences in the approach to and conceptualization of ageing. SENS considers aging from a prescriptive approach, viewing aging as not merely as a biological phenomenon but an urgent moral and political challenge. In contrast, the HoA takes a more descriptive approach, focusing on advancing the understanding of aging to provide a basis for developing and assessing interventions aimed at enhancing healthspan.

In the context of SENS, metaphors—which serve to connect complex scientific concepts with familiar experiences—play a pivotal role. Two prominent examples are the automobile maintenance metaphor and the war on aging metaphor. The former likens an organism to a car that requires regular upkeep, such as part replacement and routine inspections, to remain functional (de Grey 2008). This suggests that, much like a car, the human body could theoretically be maintained indefinitely through the repair of cellular damage, emphasizing a preventive and corrective approach to aging. Similarly, the war on aging metaphor portrays aging as a formidable adversary necessitating a strategic and coordinated response akin to a military campaign. This broad strategy has enabled SENS to attract significant funding from private tech investors and crowdfunding initiatives within the transhumanist community.

In line with their aims (see "Scope and aim" section), the communication strategies of SENS and the HoA differ not only in message content but also in their broader approach. SENS aims to reach a diverse audience, including scientists, entrepreneurs, technological investors, and advocates of radical life extension (Gems et al. 2024a). To achieve this, SENS utilizes various platforms, such as specialized scientific journals, popular science books, and public conferences (Aparicio 2025). This broad approach has enabled SENS to secure significant funding from private technological investors and crowdfunding campaigns within the transhumanist community, as emphasized by de Grey (2005c). In contrast, HoA primarily focuses on the scientific community, employing technical and specialized language that ensures precision but limits accessibility for non-experts. As a result, the HoA’s proposals are largely published in high-impact scientific journals such as *Cell* and *Nature*, with limited outreach to the general public. Their work is mainly supported by funding from public institutions, including the European Research Council (ERC) and the National Institutes of Health (NIH).

## Conclusions

Research on aging is a constantly evolving field with significant implications for health, longevity, and society at both individual and collective levels. Its primary goal is to understand the underlying biological mechanisms of aging and to develop interventions aimed at enhancing healthspan and potentially extending lifespan. Within this context, SENS and the HoA have emerged as two influential and competing frameworks, each with its advocates and critics. To clarify their proposals and delineate their similarities and differences, we have analyzed their conceptual and methodological foundations. Specifically, we have examined their scope and aims, their conceptualization of aging, their understanding of the relationship between health and disease, their approach to scientific interventions and evidence, and their communication strategies.

This analysis reveals that, from the HoA perspective, SENS may overly simplify aging by treating it merely as a purely technical problem, overlooking its broader biological complexity. Moreover, eliminating cell-molecular damage does not necessarily equate to eliminating aging, as aging might be better understood as an emergent and multifactorial process. SENS might overlook the dynamic and interconnected aspects of aging, which result not only from molecular damage but also from a complex interplay of genetic, epigenetic, and environmental factors. Conversely, from the SENS perspective, the HoA could be regarded as overly conservative, as it primarily focuses on describing aging rather than proposing concrete interventions. It may also be argued that HoA implicitly assumes aging to be an inevitable process rather than considering the possibility of reversing it through targeted biomedical strategies.

This paper seeks to contribute to a deeper understanding of the core principles and assumptions underpinning SENS and the HoA. We consider that previous literature has not sufficiently addressed the conceptual and methodological foundations of these proposals. Addressing these dimensions is essential not only for a more nuanced understanding of the frameworks themselves but also for a more accurate evaluation of their theoretical and practical contributions Such an analysis enables the identification of each approach’s strengths and limitations, while fostering critical reflection on which aspects warrant revision or refinement in future research. It may also provide a basis for assessing the prospects of a more comprehensive geroscience—one that integrates elements from multiple frameworks. Paradoxically, it may likewise open the door to exploring alternative approaches that depart fundamentally from current paradigms. We hope that this paper will stimulate further discussion on the methodological, conceptual, and philosophical foundations of contemporary research on aging.

## Data Availability

No datasets were generated or analysed during the current study.

## Footnotes

1 Currently, alternative frameworks beyond the hallmark-type framework are emerging. See, for example, Gems (2022), Gems et al. (2024b), and de Magalhães (2023; 2024).

2 The classic article that presents the main criticisms of SENS is Warner et al. (2005). In response of this article, de Grey (2005b) offered a rebuttal.

3 We thank an anonymous reviewer for encouraging us to discuss the justification and relevance of the comparative analysis.

4 Both SENS and the HoA implicitly—and more explicitly in the former—support the view that aging is undesirable and, as García-Barranquero et al. (2024a, 2024b) argue, has both direct effects on individual health and indirect consequences for the life projects people wish to pursue. However, it is worth noting that some perspectives regard aging as a process with intrinsic value and argue against biotechnological interventions aimed at altering it (Jecker 2021; Overall 2022).

5 This notion of technological solutionism within SENS, first formulated by Morozov (2013), is further explored in García-Barranquero and Bertolaso (2022) and, from a different perspective, in Porter (2023). Notably, as we will examine in "Conceptualization of aging" section, this approach defines aging not by its intrinsic biological mechanisms but by the very interventions designed to counteract it (Lemoine 2020).

6 For a comprehensive and up-to-date overview of the SENS research program, see: https://sens.org/our-research/intro-to-sens-research/

7 For a discussion of the different interpretations of whether aging should be understood as a biological or technological problem—and whether it might ultimately be solvable—see García-Barranquero and Bertolaso (2022), de Grey (2008), Hayflick (2007), Holliday (2006), Medawar (1958), and Rattan (2024).

8 For a contrary perspective, see Saborido and García-Barranquero (2022).

9 It is important to note that interventions (and their evaluation) play distinct roles in both frameworks. In SENS, interventions constitute the central and final object of discussion. In the HoA, on the other hand, interventions provide basis or criteria to address the hallmarks of aging, which themselves are the object of discussion.
